# Longitudinal Associations between Life Satisfaction and Cannabis Use Initiation, Cessation, and Disorder Symptom Severity in a Cohort of Young Swiss Men

**DOI:** 10.3390/ijerph16081372

**Published:** 2019-04-16

**Authors:** Marianthi Lousiana Deligianni, Joseph Studer, Jean-Bernard Daeppen, Gerhard Gmel, Nicolas Bertholet

**Affiliations:** 1 Addiction Medicine, Department of Psychiatry, Lausanne University Hospital and University of Lausanne, 1011 Lausanne, Switzerland; joseph.studer@chuv.ch (J.S.); jean-bernard.daeppen@chuv.ch (J.-B.D.); gerhard.gmel@chuv.ch (G.G.); nicolas.bertholet@chuv.ch (N.B.); 2 Addiction Switzerland, 1001 Lausanne, Switzerland; 3 Center for Addiction and Mental Health, Toronto, ON M6J 1H4, Canada; 4 Alcohol and Health Research Unit, University of the West of England, Bristol BS16 1QY, UK

**Keywords:** cannabis, life satisfaction, well-being, longitudinal

## Abstract

Motivations for cannabis use may include coping with negative well-being. Life satisfaction, a hallmark of subjective well-being, could play a role in cannabis use among young adults. This study aims to assess whether life satisfaction (SWLS) at age 21 is associated with cannabis initiation and cessation between the ages of 21 and 25, and with cannabis use severity (CUDIT) at age 25. Data were drawn from a cohort of young Swiss males. Associations of life satisfaction with initiation, cessation, and severity were assessed with logistic and zero-truncated negative binomial regressions. Age, family income, education, alcohol, and tobacco use at age 21 were used as adjustment variables. From a sample of 4778 males, 1477 (30.9%) reported cannabis use at age 21, 456 (9.5%) initiated use between age 21 and 25, and 515 (10.8%) ceased by age 25. Mean (SD) SWLS was significantly higher among non-users at age 21: 27.22 (5.35) vs. 26.28 (5.80), *p* < 0.001. Negative associations between life satisfaction at age 21 and cannabis use initiation (OR = 0.98, *p* = 0.029) and severity at age 25 (IRR = 0.97, *p* < 0.001) were no more significant in adjusted analyses (OR = 0.98, *p* = 0.059 and IRR = 0.99, *p* = 0.090). Life satisfaction at age 21 was not associated with cannabis cessation (OR = 0.99, *p* = 0.296). Results suggest that the predictive value of life satisfaction in cannabis use is questionable and may be accounted for by other behaviors, such as tobacco and alcohol use.

## 1. Introduction

Ongoing changes in cannabis regulation laws in recent years have raised concerns regarding its public health impact. According to the World Health Organization (WHO), cannabis is the most common illicit drug used worldwide [1]. This includes Switzerland, where cannabis prevalence is above the European average, affecting mostly those aged 15–24 [2]. Cannabis use has been associated with an increased prevalence of affective, anxiety, personality, and other substance disorders [3,4,5], as well as physical health consequences [4,5,6,7], particularly among early users during adolescence [7]. Potential detrimental effects of cannabis use include respiratory [7,8] and cognitive effects [7,9], lower educational and financial attainment [7,10], road accidents [7], risky behavior, and delinquency [4,5,11], and probably facilitates the use of other substances [7,12]. Additionally, the contribution of cannabis as a risk factor to the onset of psychosis has been widely discussed [7,9]. 

Despite its potential for harm, cannabis use is highly prevalent among adolescents and young adults [1,2]. This may be partially due to the perception that cannabis is relatively harmless in the context of a broader movement of legalization and commercialization of cannabis for recreational and medicinal purposes [13]. Motivations to use cannabis are complex and may include self-medication [14,15]. Adolescence and early adulthood are transitional periods involving academic, social, and emotional challenges; thus, cannabis may be used to cope with existential difficulties [16,17] and could be an appealing form of self-medication for individuals with lower life satisfaction.

Only a few studies have explored the relationship between life satisfaction and cannabis use [6,10,11,18,19,20,21,22,23,24,25,26], and these studies mainly used cross-sectional designs, or, when using longitudinal designs, only tested the prospective association of cannabis use on later satisfaction with life. Prospective studies showed that cannabis does not have a positive impact on satisfaction with life among young adults, but has rather the opposite effect [6,21,26]. To our knowledge, only two longitudinal studies investigated associations between life satisfaction and later cannabis use, and those results were inconsistent. A recent longitudinal study conducted in Australia suggests that lower life satisfaction during early adolescence is associated with the onset of cannabis use in young adulthood [21]. In contrast, a more recent study among disadvantaged Australians failed to support any significant associations between life satisfaction and later cannabis use [27]. Nevertheless, some authors proposed that cannabis use might be motivated by unsatisfactory life conditions [20]. Therefore, consensus is lacking regarding the influence of life satisfaction on cannabis use. Investigating the association of life satisfaction and subsequent cannabis use might reveal the processes involved in the development of cannabis use and disorders and could help in identifying individuals who may be at risk.

The aim of the present research is to fill a knowledge gap by investigating the longitudinal associations between life satisfaction at age 21 and cannabis use at age 25 in a sample of Swiss men participating in the general population-based Cohort on Substance Use Risk Factors (C-SURF). Several psychological, familial, and social factors have been associated with cannabis use onset and progression and may impact the association between life satisfaction and cannabis use. Several personality attributes, such as high levels of sensation seeking [28,29], anxiety, depression [14], and impulsivity [30] have been strongly correlated with the onset of drug use. Family and social environment variables also seem important in predicting substance use [31,32], notably having an affiliation with a group of peers who use substances [28], along with the use of alcohol and tobacco [31]. Therefore, psychological and environmental factors will be explored as potential moderators of the relationship between life satisfaction and subsequent cannabis use, especially personality and peer influence variables. 

Our primary hypothesis is that individuals with lower life satisfaction at age 21 use cannabis as a form of self-medication and that life satisfaction will be negatively associated with cannabis use initiation and subsequent cannabis use disorder, but positively associated with cannabis use cessation by age 25. We hypothesize that this association might be moderated by interpersonal and psychological variables, such as personality traits and perceived peer influence. These associations of life satisfaction and cannabis are stronger in individuals presenting vulnerable personality predispositions (i.e., high sensation seeking, neuroticism/anxiety, aggression/hospitality, and peer pressure towards misconduct and low sociability) than in individuals having less vulnerable personality predispositions and less peer pressure towards misconduct.

## 2. Materials and Methods

### 2.1. Enrollment Procedure and Participants

**Figure 1:**
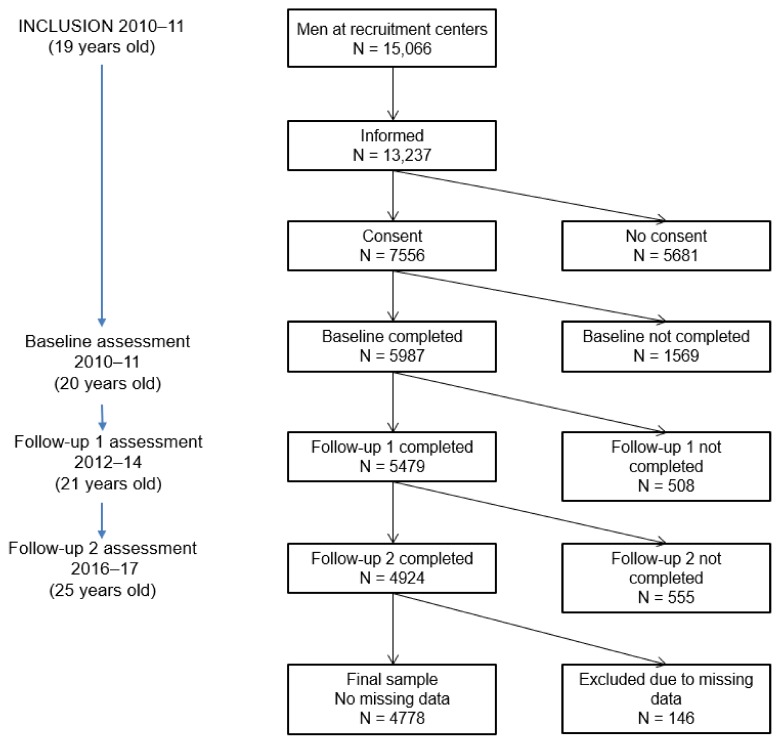
Flow chart of C-SURF participation.

C-SURF data were used for this research. Study participants were recruited between 23 August 2010 and 31 July 2011 at three of six army recruitment centers in Switzerland, which cover 21 of the 26 cantons of Switzerland. Army recruitment is mandatory in Switzerland and represents 98% of all 19-year-old males. Study procedures were independent of army involvement. Participants completed a baseline assessment at the age of 20, with follow-up assessments at age 21 and 25. C-SURF research was approved by the Ethics Committee for Clinical Research of Lausanne University Medical School (Protocol No. 15/07). A flow chart of C-SURF participation is presented in Figure 1. The sample for the present study consists of all participants who completed the baseline and two follow-up assessments.

#### Life Satisfaction

Life satisfaction was assessed at both follow-ups, using the Satisfaction with Life Scale (SWLS) [33]. It consists of five statements: “In most ways my life is close to my ideal”; “The conditions of my life are excellent”; “I am satisfied with my life”; “So far, I have gotten the important things I want in my life”; and “If I could live my life over, I would change almost nothing”. Each item is rated on a 7-point scale, from 1 (”strongly disagree”) to 7 (”strongly agree”), yielding sum scores ranging from 5 (low life satisfaction) to 35 (high life satisfaction). Cronbach’s alpha was 0.89, indicating good internal consistency of the SWLS.

### 2.2. Outcome Variables

#### Cannabis Use

Cannabis use at age 21 and age 25.

Analyses conducted within our sample [34] suggest that the number of cannabis use disorder symptoms, rather than cannabis use frequency, constitutes a more reliable measure for evaluating cannabis use. Therefore, we used cannabis use status and a validated measure, the Cannabis Use Disorder Identification Test (CUDIT) [35], to examine the associations between life satisfaction and cannabis use/cannabis use disorder severity.

Cannabis Use Status: Participants were asked whether they had used cannabis during the past 12 months. Three subgroups were created according to changes in cannabis use reported between follow-ups: those who initiated cannabis use between ages 21 and 25, those who stopped using cannabis by age 25, and those who were using cannabis at ages 21 and 25.

Cannabis Use Disorder: The CUDIT [35] was used to screen for cannabis use disorder symptoms during the past 12 months. This questionnaire contains 10 items designed to identify DSM-IV (Diagnostic and Statistical Manual of Mental Disorders, Fourth Edition) abuse and dependence, with scores ranging from zero (no misuse) to 40 (misuse).

### 2.3. Covariates

Perceived family income (below average, average, and above average), linguistic region (French- or German-speaking) at baseline, highest completed level of education (primary schooling, vocational, or post-secondary), age, alcohol use (weekly number of standard drinks), and tobacco use (none, occasional, or daily) at follow-up 1 were used to describe the sample and served as covariates in the analyses.

#### Potential Moderators

Psychological dimensions (personality traits and sensation seeking) at baseline, and peer influence at follow-up 1 were considered for potential moderators.

Personality traits: neuroticism-anxiety, sociability, and aggression-hostility traits were assessed using the cross-cultural shortened version of the Zuckerman–Kuhlman Personality Questionnaire [36]. For each subscale, participants reported whether they agreed with 10 statements (e.g., “When I get mad, I say ugly things”). Sum scores ranging from zero to 10 were computed for each of the three subscales. 

Sensation Seeking: The Brief Sensation Seeking Scale [37] was used to assess sensation seeking. Participants reported to what extent they agreed with eight statements (e.g., “I would love to have new and exciting experiences, even if they are illegal”) on a 5-point scale ranging from 1 (“strongly disagree”) to 5 (“strongly agree”). The scale score is the mean of all eight items. 

Peer Pressure: An adapted short version of the Peer Pressure Inventory [38] was used to measure perceived peer pressure towards misconduct. Participants were asked to indicate how strongly they felt pressure from friends to participate in certain activities, using five pairs of statements representing contradictory intentions (e.g., “to smoke cannabis” vs. “to not smoke cannabis”). Responses were rated on a 7-point scale ranging from −3 (“a lot of pressure to not do something”) to +3 (“a lot of pressure to do something”). The computed score is the mean of all five pairs.

### 2.4. Analyses

Logistic regressions were used to evaluate the association between life satisfaction at age 21 and initiation of cannabis use among non-users, and cessation among users. Unadjusted models (Step 1) were adjusted for demographics (Step 2) and for demographics, alcohol, and tobacco use (Step 3).

Zero-truncated negative binomial regressions assessed the association between life satisfaction at age 21 and CUDIT scores among participants using cannabis at age 21 and 25. Unadjusted models (Step 1), were then adjusted for CUDIT scores at age 21 (step 2), for CUDIT scores and demographics at age 21 (Step 3) and for CUDIT scores, demographics, alcohol, and tobacco use at age 21 (Step 4).

Although not shown in tables, four personality traits and peer influence were tested in five separate additional models (4a–e), one for each moderator. All variables, from Step 3 for logistic regressions and Step 4 for zero-truncated negative binomial regressions (as described above), were entered in models 4a–e, in addition to one moderator and the two-way interaction between moderator and life satisfaction.

Before running the analyses, multicollinearity was checked using the variance inflation factor (VIF) for each explanatory variable and interaction. No multicollinearity was detected, since the highest VIF value (all VIFs < 1.13) was well below the values (VIF = 4 or VIF = 10) that are generally considered as evidence of multicollinearity [39].

## 3. Results

### 3.1. Sample Characteristics

Table 1 shows the characteristics of the sample subgroups. From a sample of 4778 males, 3301 (69.1%) did not use cannabis at age 21, but 456 (13.8%) of the 3301 who did not use at age 21 initiated use between ages 21 and 25. Among the 1477 (30.9%) who used cannabis at age 21, 515 (34.9%) ceased using between the ages of 21 and 25. Mean (SD) SWLS was significantly higher among those who did not use cannabis at age 21 than among those who did: 27.22 (5.35) vs. 26.28 (5.80), t (4,776) = 5.48, *p* < 0.001. Mean (SD) SWL scores were 26.93 (5.51) at 21 and 26.14 (6.12) at 25. Among those using cannabis at age 21 and 25, Mean (SD) CUDIT scores were 6.66 (6.35) at age 21 and 7.01 (6.93) at age 25. Table 2 shows the prevalence of cannabis use initiation and cessation between the age of 21 and 25 by socio-demographic characteristics in the study sample.

### 3.2. Life Satisfaction and Cannabis Use Initiation 

Table 3 presents the associations of life satisfaction with cannabis use initiation among non-users at age 21. Life satisfaction at age 21 was negatively associated with cannabis initiation at age 25 (OR = 0.98, *p* = 0.029). This association remained significant after adjustment for demographic variables (OR = 0.98, *p* = 0.013), but was no longer significant when adjusted for alcohol and tobacco use (OR = 0.98, *p* = 0.059). 

### 3.3. Life Satisfaction and Cannabis Use Cessation

There was no significant association between life satisfaction at 21 and cannabis use cessation at age 25 (unadjusted: OR = 1.00, *p* = 0.987, adjusted for demographic variables: OR = 0.99, *p* = 0.617, adjusted for alcohol and tobacco use: OR = 0.99, *p* = 0.296). 

### 3.4. Life Satisfaction and CUDIT Scores

Table 4 shows the associations of life satisfaction with CUDIT scores among cannabis users at age 21 and 25. There was a negative association between life satisfaction at age 21 and CUDIT scores at age 25 (IRR = 0.97, *p* < 0.001). This remained significant after adjusting for CUDIT scores at age 21 (IRR = 0.99, *p* = 0.008), but disappeared after adjusting for demographic variables (IRR = 0.99, *p* = 0.078) and alcohol and tobacco use (IRR = 0.99, *p* = 0.090). 

## 4. Discussion

The present longitudinal study examined whether life satisfaction, a hallmark of general well-being, could predict cannabis use initiation, cessation and cannabis use disorder severity in a cohort of young Swiss males prospectively followed from the ages 21 to 25. This permitted life satisfaction to be assessed in a nonclinical, general population-based sample, both prior to and after the onset of cannabis use. During this age period of developmental challenges, prevalence rates for many types of drugs (most importantly cannabis) are the highest [1,2,16]. We hypothesized that cannabis could be used as a form of self-medication for individuals with lower life satisfaction, and therefore, life satisfaction would be negatively associated with cannabis use initiation and subsequent CUDIT scores, and positively associated with cannabis use cessation. Consistent with our hypothesis, life satisfaction at age 21 was negatively associated with cannabis use initiation and severity in this sample; however, the association lost significance in adjusted analyzes for demographics, alcohol, and tobacco use. This suggests that the association may be accounted for by differences in these variables. The association between low life satisfaction and cannabis use initiation appears to be largely influenced by confounding factors. Previous longitudinal studies [21,27] have suggested that a drop in life satisfaction preceded cannabis use initiation and failed to report the same impact of any potential confounding factors. This may be explained by differences in their target population (mostly adolescents or disadvantaged groups) and in the instruments used (no validated measure of life satisfaction and different measures of cannabis use).

Another possible explanation for the lack of significance may be that the influence of life satisfaction does not last long enough to be captured in a follow-up span of four years.

In our sample, life satisfaction was assessed using the extensively validated satisfaction with life scale (SWLS) that has been shown to correlate with mental health and future behaviors [40,41]. Cannabis users and non-users alike were generally satisfied with life [40], although mean (SD) SWLS scores were significantly higher among non-users than among users at age 21: 27.22 (5.35) vs. 26.28 (5.80) for users, t (4776) = 5.48, *p* < 0.001. Mean (SD) SWL scores were 26.93 (5.51) at 21 and 26.14 (6.12) at 25. This is consistent with observations from other non-clinical populations [41]. Similar scores were observed among young adults in recent studies showing negative correlations between life satisfaction and cannabis use frequency [6,20]. These scores are higher than were those indicated in an earlier study evaluating life satisfaction among daily cannabis users aged 18–88 [42].

It is important to note that among the 962 participants using cannabis at the ages of 21 and 25, there were only 157 (16.3%) that did not use tobacco. This is consistent with existing evidence indicating that up to 90% of cannabis users have concomitant tobacco use [43]. Epidemiological evidence supports common underlying mechanisms of concomitant use [43,44], with tobacco use often preceding cannabis use initiation [45]. In our analyses, associations between life satisfaction and cannabis use initiation were no longer significant after adjustment for alcohol and tobacco use, with the effect of tobacco being more prominent. This is consistent with the involvement of tobacco use in cannabis use initiation [12]. Another possible interpretation is that tobacco might be more appealing (or more readily available) than cannabis as a way to cope with unsatisfactory or unpleasant life conditions. It is important to note that during the study period, cannabis was illegal in Switzerland, thus affecting its accessibility within our cohort. Similarly, to what we observed regarding cannabis initiation, associations between life satisfaction and CUDIT were no longer significant in adjusted analyses; the effect was predominantly accounted for by daily tobacco use. This is consistent with findings from previous studies suggesting that tobacco use is a key driver in cannabis use disorder development and severity [44,46].

Previous studies on life satisfaction and health behaviors have demonstrated negative associations between life satisfaction and tobacco use. A cross-cultural analysis of 17,246 students aged 17–30 years from 21 countries found that individuals with higher life satisfaction were less likely to smoke [47]. Similarly, a national Finnish study found significant associations between life dissatisfaction and adverse health behaviors, most notably heavy drinking and smoking [48]. 

This study has several limitations. First, the study sample consists of young men only. Therefore, results cannot be generalized to women and other age groups. Second, all data rely on self-report without biochemical verification. Underreporting of substance use is therefore possible because of social desirability or fear of stigma. Nonetheless, the study has some notable strengths: namely, the use of validated instruments to assess life satisfaction and cannabis use, low attrition, and a large sample of cohorts gathered from geographically and culturally diverse backgrounds.

## 5. Conclusions

In contrast to the findings of previous studies, our results suggest that the predictive value of life satisfaction in cannabis use initiation is questionable and may be accounted for mostly by other behaviors such as tobacco and alcohol use, indicating that the relationships found previously may be due to various confounding factors. However, this does not exclude the possibility that life satisfaction remains a factor underlying substance use in general. Indeed, other more readily available substances, such as tobacco, are probably more likely to be used for coping with unpleasant or unsatisfactory life conditions. Future research on life satisfaction determinants among substance users, and particularly among young tobacco users, may elucidate potential associations and provide novel targets for interventions. If negative well-being does actually precede health risk behaviors such as substance use, then the implementation of well-being enhancing strategies among at risk populations may prove useful in substance use prevention policies.

Nevertheless, our results do not support the hypothesis that life satisfaction plays a predominant role in contributing to either the initiation or the severity of cannabis use.

## Figures and Tables

**Table 1:** Descriptive characteristics of the study sample (N = 4778).

	CANNABIS USE AT AGE 21											
	NO						YES					
	Total N = 3301		CANNABIS USE AT AGE 25				Total N = 1477		CANNABIS USE AT AGE 25			
			Yes N = 456 (i.e., Initiation of Cannabis Use)		No N = 2845				Yes N = 962		No N = 515 (i.e., Cessation of Cannabis Use)	
	Mean/N	SD/%	Mean/N	SD/%	Mean/N	SD/%	Mean/N	SD/%	Mean/N	SD/%	Mean/N	SD/%
Life satisfaction	27.22	5.35	26.71	5.69	27.30	5.29	26.28	5.80	26.28	5.79	26.27	5.83
Age at FU1	21.28	1.26	21.09	1.15	21.31	1.27	21.26	1.25	21.26	1.25	21.26	1.25
Drinking volume	6.22	8.16	7.64	7.99	5.99	8.16	11.66	10.19	12.27	10.00	10.52	10.44
Aggression-hostility	3.99	2.21	4.28	2.16	3.94	2.22	4.40	2.21	4.45	2.19	4.30	2.24
Sociability	5.73	2.29	5.88	2.17	5.71	2.31	6.09	2.13	6.14	2.13	5.98	2.15
Anxiety-neuroticism	1.89	1.92	2.05	2.03	1.86	1.91	2.10	2.11	2.07	2.11	2.17	2.10
Sensation seeking	2.89	0.84	3.14	0.82	2.85	0.84	3.41	0.78	3.47	0.77	3.28	0.77
Peer pressure	−0.09	0.63	0.05	0.57	−0.12	0.64	0.28	0.51	0.32	0.51	0.20	0.50
Family income												
Below average	448	13.6	65	14.3	383	13.5	196	13.3	125	13.0	71	13.8
Average	1403	42.5	203	44.5	1200	42.2	587	39.7	389	40.4	198	38.4
Above average	1450	43.9	188	41.2	1262	44.4	694	47.0	448	46.6	246	47.8
Highest level of education												
Primary schooling	245	7.4	34	7.5	211	7.4	134	9.1	101	10.5	33	6.4
Vocational	1397	42.3	166	36.4	1231	43.3	489	33.1	303	31.5	186*	36.1
Post-secondary	1659	50.3	256	56.1	1403	49.3	854	57.8	558	58.0	296	57.5
Language												
French	1857	56.3	255	55.9	1602	56.3	827	56.0	549	57.1	278	54.0
German	1444	43.7	201	44.1	1243	43.7	650	44.0	413	42.9	237	46.0
Cigarette smoking												
No	2260	68.5	239	52.4	2021	71.0	308	20.9	157	16.3	151	29.3
Occasional	633	19.2	139	30.5	494	17.4	620	42.0	415	43.1	205	39.8
Daily	408	12.4	78	17.1	330	11.6	549	37.2	390	40.5	159	30.9

**Table 2:** Prevalence of cannabis use initiation and cessation between the age of 21 and 25 in the study sample.

Initiation at Age 25 (Among Non-Users at Age 21, N = 3301) %		Cessation at Age 25 (Among Users at age 21, N = 1477) %
Family income		
Below Average	14.5	36.2
Average	14.5	33.7
Above average	13.0	35.5
Highest level of education		
Primary schooling	13.9	24.6
Vocational	11.9	38.0
Post-secondary	15.4	34.7
Language		
French	13.7	33.6
German	13.9	36.5
Cigarette smoking		
No	10.6	49.0
Occasional	22.0	33.0
Daily	19.1	29.0
Full sample	13.8	34.9

**Table 3:** Life satisfaction at age 21 (Step 1), demographics (Step 2), and alcohol and tobacco use (Step 3) predicting initiation and cessation of cannabis use at age 25 (logistic regressions).

	OR (95% CI)	
Life Satisfaction at 21 and cannabis Use at 25	Initiation	Cessation
**Step 1: Bivariate**		
Life satisfaction at age 21	0.98 (0.96;1.00) *^a^	1.00 (0.98;1.02)
**Step 2: Adjusted for demographics**		
Life satisfaction at age 21	0.98 (0.96;0.99) *	0.99 (0.98;1.01)
Family income (ref. below average)		
Average	1.03 (0.76;1.41)	0.90 (0.64;1.27)
Above average	0.86 (0.63;1.18)	0.96 (0.68;1.34)
Highest level of education (ref. Primary schooling)		
Vocational	0.94 (0.62;1.41)	1.89 (1.22;2.95) *
Post-secondary	1.29 (0.86;1.92)	1.67 (1.08;2.58) *
Linguistic region (ref. German-speaking)		
French-speaking	1.00 (0.81;1.25)	0.92 (0.73;1.16)
Age	0.86 (0.78;0.94) ***	1.01 (0.92;1.10)
**Step 3: Adjusted for demographics and alcohol and tobacco use**		
Life satisfaction at age 21	0.98 (0.96;1.00)	0.99 (0.97;1.01)
Family income (ref. below average)		
Average	1.02 (0.75;1.40)	0.91 (0.64;1.29)
Above average	0.84 (0.61;1.16)	0.94 (0.67;1.33)
Highest level of education (ref. Primary schooling)		
Vocational	0.89 (0.59;1.35)	1.89 (1.21;2.96) *
Post-secondary	1.41 (0.94;2.11)	1.50 (0.97;2.34)
Linguistic region (ref. German-speaking)		
French-speaking	0.98 (0.78;1.22)	0.91 (0.72;1.15)
Age	0.86 (0.78;0.94) **	1.02 (0.93;1.12)
Drinking volume	1.01 (1.00;1.02) *^b^	0.99 (0.97;1.00) *^c^
Smoking status (ref. non-smoker)		
Occasional smoker	2.30 (1.81;2.91) ***	0.54 (0.40;0.71) **
Daily smoker	2.15 (1.60;2.89) ***	0.43 (0.31;0.58) **

*Note.* OR = Odds Ratio. CI = Confidence Interval. ^a^ Before rounding, higher bound of 95% CI = 0.997922. ^b^ Before rounding, lower bound of 95% CI = 1.002454. ^c^ Before rounding, higher bound of 95% CI = 0.998061. * *p* < 0.05; ** *p* < 0.01; *** *p* < 0.001.

**Table 4:** Life satisfaction at age 21 (Step 1), CUDIT score at age 21 (step 2), demographics (Step 3), and alcohol and tobacco use (Step 4) predicting CUDIT scores at age 25 (Zero-truncated negative binomial regressions).

Life Satisfaction at 21 and CUDIT Score at 25	IRR (95% CI)
**Step 1: Bivariate**	
Life satisfaction at age 21	0.97 (0.96;0.98) ***
**Step 2: Adjusted for CUDIT at age 21**	
Life satisfaction at age 21	0.99 (0.97;1.00) **^a^
CUDIT score at age 21	1.11 (1.10;1.12) ***
**Step 3: Adjusted for CUDIT at age 21 and demographics**	
Life satisfaction at age 21	0.99 (0.98;1.00)
CUDIT score at age 21	1.11 (1.10;1.12) ***
Family income (ref. below average)	
Average	1.13 (0.92;1.39)
Above average	1.04 (0.85;1.27)
Highest level of education (ref. Primary schooling)	
Vocational	0.97 (0.78;1.22)
Post-secondary	0.83 (0.67;1.03)
Linguistic region (ref. German-speaking)	
French-speaking	0.95 (0.82;1.09)
Age	1.06 (1.00;1.12) *^b^
**Step 4: Adjusted for CUDIT at age 21 and demographics and alcohol and tobacco use**	
Life satisfaction at age 21	0.99 (0.98;1.00)
CUDIT score at age 21	1.10 (1.09;1.11) ***
Family income (ref. below average)	
Average	1.12 (0.92;1.38)
Above average	1.07 (0.88;1.31)
Highest level of education (ref. Primary schooling)	
Vocational	0.97 (0.77;1.20)
Post-secondary	0.88 (0.71;1.10)
Linguistic region (ref. German-speaking)	
French-speaking	0.97 (0.85;1.11)
Age	1.05 (1.00;1.11)
Drinking volume	1.00 (0.99;1.01)
Smoking status (ref non-smoker)	
Occasional smoker	1.08 (0.89;1.31)
Daily smoker	1.46 (1.20;1.78) ***

*Note.* IRR = Incidence Rate Ratio. CI = Confidence Interval. ^a^ Before rounding, higher bound of 95% CI = 0.9962147. ^b^ Before rounding, lower bound of 95% CI = 1.004827. * *p* < 0.05; ** *p* < 0.01; *** *p* < 0.001.

## References

[1] WHO (2016). The Health and Social Effects of Nonmedical Cannabis Use.

[2] Gmel G., Kuendig H., Notari L., Gmel C. (2017). Monitorage Suisse des Addictions: Consommation D’alcool, Tabac et Drogues Illégales en Suisse en 2016.

[3] Hasin D.S., Kerridge B.T., Saha T.D., Huang B., Pickering R., Smith S.M., Jung J., Zhang H., Grant B.F. (2016). Prevalence and correlates of DSM-5 cannabis use disorder, 2012–2013: Findings from the national epidemiologic survey on alcohol and related conditions-III. Am. J. Psychiatry.

[4] Bruno A., Scimeca G., Marino A.G., Mento C., Micò U., Romeo V.M., Pandolfo G., Zoccali R., Muscatello M.R. (2012). Drugs and Sexual Behavior. J. Psychoact. Drugs.

[5] Scimeca G., Chisari C., Muscatello M.R.A., Cedro C., Pandolfo G., Zoccali R., Bruno A. (2017). Cannabis and Sexual Behavior. Handb. Cannabis Relat. Pathol..

[6] Arria A.M., Caldeira K.M., Bugbee B.A., Vincent K.B., O’Grady K.E. (2016). Marijuana use trajectories during college predict health outcomes nine years post-matriculation. Drug Alcohol Depend..

[7] Hall W. (2015). What has research over the past two decades revealed about the adverse health effects of recreational cannabis use?. Addiction.

[8] Reid P.T., Macleod J., Robertson J.R. (2010). Cannabis and the lung. J. R. Coll. Physicians Edinb..

[9] Volkow N.D., Swanson J.M., Evins A.E., De Lisi L.E., Meier M.H., Gonzalez R., Bloomfield M.A., Curran H.V., Baler R. (2016). Effects of Cannabis Use on Human Behavior, Including Cognition, Motivation, and Psychosis: A Review. JAMA Psychiatry.

[10] Gruber A.J., Pope H.G., Hudson J.I., Yurgelun-Todd D. (2003). Attributes of long-term heavy cannabis users: A case-control study. Psychol. Med..

[11] Fergusson D.M., Boden J.M. (2008). Cannabis use and later life outcomes. Addiction.

[12] Haug S., Núñez C.L., Becker J., Gmel G., Schaub M.P. (2014). Predictors of onset of cannabis and other drug use in male young adults: Results from a longitudinal study. BMC Public Health.

[13] Stolzenberg L., D’Alessio S.J., Dariano D. (2016). The effect of medical cannabis laws on juvenile cannabis use. Int. J. Drug Policy.

[14] Wittchen H.-U., Fröhlich C., Behrendt S., Günther A., Rehm J., Zimmermann P., Lieb R., Perkonigg A. (2007). Cannabis use and cannabis use disorders and their relationship to mental disorders: A 10-year prospective-longitudinal community study in adolescents. Drug Alcohol Depend..

[15] Leventhal A.M., Cho J., Stone M.D., Barrington-Trimis J.L., Chou C.P., Sussman S.Y., Riggs N.R., Unger J.B., Audrain-McGovern J., Strong D.R. (2017). Associations between anhedonia and marijuana use escalation across mid-adolescence. Addiction.

[16] Arnett J.J. (2005). The Developmental Context of Substance use in Emerging Adulthood. J. Drug Issues.

[17] Moitra E., Christopher P.P., Anderson B.J., Stein M.D. (2015). Coping-motivated marijuana use correlates with DSM-5 cannabis use disorder and psychological distress among emerging adults. Psychol. Addict. Behav..

[18] White H.R., Bechtold J., Loeber R., Pardini D. (2015). Divergent marijuana trajectories among men: Socioeconomic, relationship, and life satisfaction outcomes in the mid-30s. Drug Alcohol Depend..

[19] Ellickson P.L., Martino S.C., Collins R.L. (2004). Marijuana Use from Adolescence to Young Adulthood: Multiple Developmental Trajectories and Their Associated Outcomes. Health Psychol..

[20] Tartaglia S., Miglietta A., Gattino S. (2017). Life Satisfaction and Cannabis Use: A Study on Young Adults. J. Happiness Stud..

[21] Fischer J.A., Clavarino A.M., Plotnikova M., Najman J.M. (2015). Cannabis Use and Quality of Life of Adolescents and Young Adults: Findings from an Australian Birth Cohort. J. Psychoact. Drugs.

[22] Barnwell S.S., Earleywine M., Wilcox R. (2006). Cannabis, motivation, and life satisfaction in an internet sample. Subst. Abus. Treat. Prev. Policy.

[23] Swain N.R., Gibb S.J., Horwood L.J., Fergusson D.M. (2012). Alcohol and cannabis abuse/dependence symptoms and life satisfaction in young adulthood. Drug Alcohol Rev..

[24] Bogart L.M., Collins R.L., Ellickson P.L., Klein D.J. (2007). Are adolescent substance users less satisfied with life as young adults and if so, why?. Soc. Indic. Res..

[25] Zullig K.J., Valois R.T., Huebner E., Oeltmann J.E., Drane W. (2001). Relationship between perceived life satisfaction and adolescents’ substance abuse. J. Adolesc. Health.

[26] Allen J., Holder M.D. (2014). Marijuana Use and Well-Being in University Students. J. Happiness Stud..

[27] Moschion J., Powdthavee N. (2018). The welfare implications of addictive substances: A longitudinal study of life satisfaction of drug users. J. Econ. Behav. Organ..

[28] Creemers H.E., Dijkstra J.K., Vollebergh W.A.M., Ormel J., Verhulst F.C., Huizink A.C. (2010). Predicting life-time and regular cannabis use during adolescence; The roles of temperament and peer substance use: The TRAILS study. Addiction.

[29] Foulds J.A., Boden J.M., Newton-Howes G.M., Mulder R.T., Horwood L.J. (2017). The role of novelty seeking as a predictor of substance use disorder outcomes in early adulthood. Addiction.

[30] Hayatbakhsh M.R., McGee T.R., Bor W., Najman J.M., Jamrozik K., Mamun A.A. (2008). Child and adolescent externalizing behavior and cannabis use disorders in early adulthood: An Australian prospective birth cohort study. Addict. Behav..

[31] Agrawal A., Lynskey M.T. (2009). Correlates of later-onset cannabis use in the National Epidemiological Survey on Alcohol and Related Conditions (NESARC). Drug Alcohol Depend..

[32] Hayatbakhsh R., Williams G.M., Bor W., Najman J.M. (2013). Early childhood predictors of age of initiation to use of cannabis: A birth prospective study. Drug Alcohol Rev..

[33] Diener E., Emmons R., Larsen J., Griffin S. (1985). The satisfaction with life scale. J. Pers. Assess..

[34] Baggio S., N’Goran A.A., Deline S., Studer J., Dupuis M., Henchoz Y., Mohler-Kuo M., Daeppen J.B., Gmel G. (2014). Patterns of cannabis use and prospective associations with health issues among young males. Addiction.

[35] Adamson S.J., Sellman J.D. (2003). A prototype screening instrument for cannabis use disorder: The Cannabis Use Disorders Identification Test (CUDIT) in an alcohol-dependent clinical sample. Drug Alcohol Rev..

[36] Aluja A., Rossier J., García L.F., Angleitner A., Kuhlman M., Zuckerman M. (2006). A cross-cultural shortened form of the ZKPQ (ZKPQ-50-cc) adapted to English, French, German, and Spanish languages. Pers. Individ. Dif..

[37] Hoyle R.H., Stephenson M.T., Palmgreen P., Lorch E.P., Donohew R.L. (2002). Reliability and validity of a brief measure of sensation seeking. Pers. Individ. Dif..

[38] Baggio S., Studer J., Daeppen J.-B., Gmel G. (2013). Adaptation of a peer pressure scale in French and German: The Peer Pressure Inventory. Rev. Epidemiol. Sante Publique.

[39] O’Brien R.M. (2007). A caution regarding rules of thumb for variance inflation factors. Qual. Quant..

[40] Pavot W., Diener E. (1993). Review of the Satisfaction With Life Scale. Psychol. Assess..

[41] Pavot W., Diener E. (2008). The Satisfaction With Life Scale and the emerging construct of life satisfaction. J. Posit. Psychol..

[42] Looby A., Earleywine M. (2007). Negative consequences associated with dependence in daily cannabis users. Subst. Abus. Treat. Prev. Policy.

[43] Agrawal A., Budney A.J., Lynskey M.T. (2012). The co-occurring use and misuse of cannabis and tobacco: A review. Addiction.

[44] Rabin R.A., George T.P. (2015). A review of co-morbid tobacco and cannabis use disorders: Possible mechanisms to explain high rates of co-use. Am. J. Addict..

[45] Kandel D. (1975). Stages in adolescent involvement in drug use. Science.

[46] Hindocha C., Shaban N.D.C., Freeman T.P., Das R.K., Gale G., Schafer G., Falconer C.J., Morgan C.J., Curran H.V. (2015). Associations between cigarette smoking and cannabis dependence: A longitudinal study of young cannabis users in the United Kingdom. Drug Alcohol Depend..

[47] Grant N., Wardle J., Steptoe A. (2009). The relationship between life satisfaction and health behavior: A cross-cultural analysis of young adults. Int. J. Behav. Med..

[48] Koivumaa-Honkanen H., Honkanen R., Viinamäki H., Heikkilä K., Kaprio J., Koskenvuo M. (2000). Self-reported life satisfaction and 20-year mortality in healthy finnish adults. Am. J. Epidemiol..

